# Emerging Sponge Models of Animal-Microbe Symbioses

**DOI:** 10.3389/fmicb.2016.02102

**Published:** 2016-12-23

**Authors:** Lucia Pita, Sebastian Fraune, Ute Hentschel

**Affiliations:** ^1^RD3 Marine Microbiology, GEOMAR Helmholtz Centre for Ocean ResearchKiel, Germany; ^2^Zoological Institute, Christian-Albrechts-University of Kiel (CAU), KielGermany; ^3^Christian-Albrechts-University of Kiel (CAU), KielGermany

**Keywords:** Porifera, holobiont, microbiota, aquaculture, metazoan symbiosis, RNAi, CRISPR-Cas

## Abstract

Sponges have a significant impact on marine benthic communities, they are of biotechnological interest owing to their production of bioactive natural compounds, and they promise to provide insights into conserved mechanisms of host–microbe interactions in basal metazoans. The natural variability of sponge-microbe associations across species and environments provides a meaningful ecological and evolutionary framework to investigate animal-microbial symbiosis through experimentation in the field and also in aquaria. In addition, next-generation sequencing technologies have shed light on the genomic repertoire of the sponge host and revealed metabolic capacities and symbiotic lifestyle features of their microbiota. However, our understanding of symbiotic mechanisms is still in its infancy. Here, we discuss the potential and limitations of the sponge-microbe symbiosis as emerging models for animal-associated microbiota.

## Introduction

Each model of animal-associated microbiota offers unique opportunities to address questions related to symbiosis (Ruby, 2008; Bosch and McFall-Ngai, 2011; Kostic et al., 2013). Existing laboratory models, such as fly (*Drosophila melanogaster*), worm (*Caenorhabditis elegans*), zebra fish or mice, enable to test the influence of genetic or environmental factors on symbioses. However, it remains unclear to which extent the findings in lab models apply to natural systems and to other taxa. Natural models provide a relevant ecological and evolutionary framework, but are frequently restricted to systems in which the number of players and interactions is reduced (e.g., the squid *Euprymna scolopes* and the bacterium *Vibrio fischeri*, or the mussels *Bathymodiolus* spp. and their microbiota). However, owing to deep-sequencing technologies, it has become clear that many animals are associated with complex microbial consortia and the implications of such symbioses are just beginning to be unraveled (McFall-Ngai et al., 2013). The study of complex consortia is challenging because multiple interactions take place simultaneously, making it difficult to decipher the specific roles of each symbiont. Additionally, methodologies are limiting and microbes are frequently recalcitrant to cultivation. Enhancing the tractability of the symbiosis within different animal phyla would contribute to our understanding on animal–microbe interactions.

Marine sponges (phylum Porifera) represent prominent examples for such complex symbioses. Many sponge species contain diverse microbial consortia within their mesohyl matrix that can reach densities of up to 10^9^ microbial cells/cm^3^ of sponge (Hentschel et al., 2006). Sponges belong to a phylum that originated ca. 600 million years ago (Li et al., 1998). Their porous body plan contains a highly ramified aquiferous canal system through which seawater is pumped. Specific cells lining the choanocyte chambers (termed “choanocytes”) take up particles, such as bacterioplankton, from the seawater and transfer them into the mesohyl interior where these are digested by phagocytosis. Sponges lack organs, muscles, and a nervous system. In spite of their simple anatomy, recent studies have revealed an unexpected genomic complexity in sponges (Srivastava et al., 2010; Riesgo et al., 2014a). For example, they express homologs of genes involved in the animal nervous system (Ludeman et al., 2014) and possess central elements of the Toll-like receptor signaling cascade, potentially involved in innate immunity (Hentschel et al., 2012; Riesgo et al., 2014a). In addition, sponges and their associated microorganisms yield secondary metabolites that are of relevance to biotechnological and medical applications (Mehbub et al., 2014; Indraningrat et al., 2016).

The sponge-associated microbiota is exceedingly complex with thousands of symbiont lineages reported per sponge individual (Thomas et al., 2016). The most abundant phyla are *Proteobacteria, Chloroflexi*, and *Crenarchaeota*, among others. Altogether, more than 40 microbial phyla, including several candidate phyla (e.g., *Tectomicrobia, Poribacteria*) were recovered from sponges. In contrast, most animal-associated microbiota belong within 3–5 phyla and diversification is found at species and strain level (Kostic et al., 2013). Some members of the sponge-associated consortia are vertically transmitted to the next generation by larval stages (De Caralt et al., 2007b; Schmitt et al., 2008), but horizontal acquisition also appears likely (Taylor et al., 2007). Sponge symbiont OTUs have also been recovered from seawater, albeit at very low abundances, and their activity in the free-living state still needs to be explored (Webster et al., 2010). The diversity patterns, metabolic repertoire and genomic features of sponge-associated microbiota have been reviewed in detail elsewhere (Taylor et al., 2007; Hentschel et al., 2012; Webster and Thomas, 2016). Despite a species-specific composition, similar functions are detected in the microbiomes of distantly related sponge species, suggesting convergent evolution (Fan et al., 2012; Ribes et al., 2012; Thomas et al., 2016; Horn et al., 2016). These functions relate to nutritional interactions (e.g., nitrification, vitamin B synthesis), host–microbe recognition [e.g., eukaryotic-like proteins (ELPs)] and adaptation to host’s internal environment (e.g., CRISPR-Cas defense system).

A significant body of information has been accrued from analyzing the natural variability of sponge microbiomes in different host species and environments. Host species appears to be the main factor driving microbial diversity (Erwin et al., 2012; Easson and Thacker, 2014), although environmental factors (e.g., intertidal vs. subtidal habitat, Weigel and Erwin, 2016; depth, Steinert et al., 2016; location, Pita et al., 2013b) can also cause intraspecific variability. Also, differences in symbiont density within the mesohyl (i.e., high microbial abundance HMA sponges vs. low microbial abundance LMA sponges; Gloeckner et al., 2014) have an impact on microbial community composition as well as on the host pumping rate and other metabolic parameters (Weisz et al., 2008; Ribes et al., 2012). Monitoring microbiota changes over seasons, bleaching episodes, natural gradients, or upon transplantation showed the plasticity of the symbiosis at scales that are difficult to mimic in laboratory (Steindler et al., 2007; López-Legentil et al., 2010; Erwin et al., 2015; Morrow et al., 2015). As similar past and present environment has been also faced by other benthic invertebrates, the features and mechanisms of sponge-microbe symbioses contribute to a more comprehensive view of marine symbiotic systems.

The need for sponge models for symbiosis has been debated within the sponge microbiology community (i.e., at the 1st International Symposium of Sponge Microbiology, Taylor et al., 2011). While the benefits of pooling resources, developing standardized protocols and limiting redundancy were clearly acknowledged, the dangers of developing too narrow a view of the natural diversity were also voiced (Taylor et al., 2011; Webster and Taylor, 2012). An experimental sponge model would, however, be immensely useful to put the large amount of sequence data into functional context. Even though some advances toward an experimental sponge model have been made, such as the generation of protocols and procedures for sponge aquaculture (Schippers et al., 2012) as well as the silencing of sponge genes for functional studies (Rivera et al., 2011), the overall efforts are still in its infancy. Here we discuss the current status and future directions toward establishing an experimental sponge model for symbiosis.

## Desired Properties for an Experimental Sponge Model

Disclosing the mechanistic bases of sponge–microbe interactions requires experimental tractability. The desired features of an experimental sponge model are depicted in **Table 1**. First, species that are accessible to as many laboratories as possible would be useful candidates. Most studied sponge species thrive in shallow temperate or tropical waters, where they are easily collected by snorkeling or scuba diving. Sponges from remote or deep environments in need of ocean-going equipment, expertise, and resources are naturally less amenable to experimental manipulation. Sponge species inhabiting a wide geographic range should be prioritized; however, many species are limited to distinct geographic regions. Consequently, most research groups have focused on the abundant species at their local sites (e.g., the Great Barrier Reef species *Amphimedon queenslandica, Rhopaloides odorabile, Cymbastela concentrica*, the West-Atlantic/Caribbean/East-Pacific sponges *Xestospongia muta, Mycale laxissima, Axinella mexicana*, or the Mediterranean/East-Atlantic species *Aplysina aerophoba, Petrosia ficiformis, Dysidea avara, Ircinia s*pp.), thus making an overall comparison across geographic boundaries difficult. On the genus level, some sponges show an almost global distribution within temperate and/or tropical latitudes (e.g., *Aplysina, Halichondria, Ircinia*, and *Xestospongia*) and laboratories in different geographic areas would benefit from protocols applicable to these sponges. In addition, when choosing a model system, the parameters of morphology and size should be taken into account. Smaller, compact species are easier to collect and maintain than massive or fragile species. For example, while *Xestospongia muta* satisfies the criteria of wide distribution and ecological relevance, it will hardly be amenable to aquarium maintenance owing to its large size and fragility.

**Table 1:** Desired properties for an experimental sponge model for symbiosis (adapted after Ruby, 2008).

• Ease of collection, wide geographic distribution
• Simple morphology, small size
• Maintenance in aquaculture
• Closed life cycle
• Establishment of a gnotobiotic/aposymbiotic host
• Availability of omic-data
• Amenability to genetic manipulation (RNAi, CRISPR-Cas)
• Culturability of symbionts

An experimental model requires the long-term controlled maintenance of the holobiont (here defined as the host plus its symbionts, Margulis, 1991). Different laboratories have kept sponges in aquaria for days/weeks (e.g., Fan et al., 2013; Pita et al., 2013a), several months (e.g., *Aplysina aerophoba*, Gerçe et al., 2009; Sacristán-Soriano et al., 2016), and up to 2 years (*Ircinia strobilina*, Mohamed et al., 2008b; *Mycale laxissima*, Mohamed et al., 2008a). The aquaculture setups vary from closed systems with external food supply (i.e., bacteria or algae) to open flow-through systems with direct seawater uptake, which are closer to *in situ* conditions but demand advanced infrastructure. Depending on the sponge species and/or the aquaculture system, the microbiota changes upon transfer and long-term maintenance in aquaria with respect to community composition and abundance, although the core microbiome seems to persist (e.g., Mohamed et al., 2008a; Webster et al., 2011; Ribes et al., 2016). This plasticity requires careful monitoring of the microbial diversity during aquaculture by use of standardized protocols (Earth Microbiome Project, Thomas et al., 2016).

Thus far, sponge experimentation in aquaria depends on the collection of specimens from the field and their transfer to the lab. The limitations to *in vitro* reproduction of sponges are mainly due to their life cycle as the reproductive period typically occurs once a year, because reproductive cues vary among species (Riesgo and Maldonado, 2008), and because juveniles have higher mortality rates in aquaria (De Caralt et al., 2007a). It is therefore noteworthy, that the life cycles of the Mediterranean sponges *Dysidea avara* and *Crambe crambe* and of the Australian sponge *Amphimedon queenslandica* were successfully closed *in vitro* (De Caralt et al., 2007a; Conaco et al., 2012). In addition to field collections, clonal populations of sponges can be generated and maintained owing to sponge reproduction by gemmulation or budding (Adams et al., 2010; Di Bari et al., 2015). The quick regeneration capacity after fragmentation can be used to generate “explants,” which are clonal pieces of sponge that will continue to be metabolically active in aquaria. Such efforts were undertaken with the species *Halichondria panicea* (Barthel and Theede, 1986), *Corticium candelabrum* (De Caralt et al., 2003), *Geodia barretti* (Hoffmann et al., 2003), and *Rhopaloides odorabile* (Webster et al., 2011) and have provided new insights into primary metabolism of the sponge holobiont. The generation of sponge fragments may be useful in the future to rear sponge individuals of similar/identical genetic background at small sizes and with a high number of replicates.

Aposymbiotic states (i.e., deprivation of a particular group of symbionts) and germ-free hosts are crucial tools to dissect the animal-microbiota crosstalk. The most common method to rear germ-free animals is the application of antibiotics. However, the sponge microbiota appears to be resistant to antibiotics (Friedrich et al., 2001; De Caralt et al., 2003), even when applied to sponge cell aggregates (Richardson et al., 2012). Interestingly, aposymbiotic sponges occur in the field. The Mediterranean sponge *Petrosia ficiformis* harbors the cyanobacterium *Synechococcus feldmannii*; however, individuals in caves appear photosymbiont-free (Burgsdorf et al., 2014). Also, the Caribbean sponge *Xestospongia muta* undergoes cyclic bleaching – density loss of symbiont *Synechococcus* spp. –, from which the sponge can recover (López-Legentil et al., 2008). If the cue for aposymbiosis is discerned, it could potentially be simulated in the lab. In a different approach, Riesgo et al. (2014b) created dinoflagellate-free *Cliona varians* specimens by removing the outer layer of the sponges, where the photosymbionts thrive. Aposymbiotic *Cliona varians* sponges were able to recapture photosymbionts. Unfortunately, the available examples are still restricted to few species and the photosynthetic symbionts. Alternative approaches based on phage therapy, a clinical strategy to clean infections and tumoral cells (e.g., Cattaneo et al., 2008; Nobrega et al., 2015), remain unexplored but are promising tools to selectively remove symbionts.

In the last 5 years, the amount of genetic information available on sponge hosts in the context of symbiosis has increased exponentially (**Table 2**). The first published genome – from *A. queenslandica*, with a size of 166.7 Mb and mean GC percentage of 37.5% (Srivastava et al., 2010)– is still the reference for Porifera. Recently, the enhanced annotation of this genome raised the total number of genes from 28898 (first version, Srivastava et al., 2010) to 40122 (Fernandez-Valverde et al., 2015). This finding suggests a more compacted genome than previous thought, but also reflects the difficulties of identifying the structure and function of genes in species that are phylogenetically distant from traditional model organisms. Even the role of genes with known homologs in other phyla needs to be confirmed in experimental studies in this group. As Porifera is a highly diversified phylum, the genetic information of different species is necessary to gain a more comprehensive view of the sponge genetic toolkit (Riesgo et al., 2014a).

**Table 2:** A compilation of sponge species for which published omic-data in the context of the sponge symbiosis are available.

Sponge Species	General properties	Existing omic data
*Amphimedon queenslandica*	Collection: Australia Microbial association: LMA	Sponge genome (Srivastava et al., 2010) Sponge transcriptome (Conaco et al., 2012; Fernandez-Valverde et al., 2015)
*Aplysina aerophoba*	Collection: Mediterranean Sea Microbial association: HMA	Microbial metagenome (Horn et al., 2016) Symbiont genomes: *Poribacteria* (Siegl et al., 2011; Kamke et al., 2014); *Synechococcus spongiarum* (Burgsdorf et al., 2015)
*Carteriospongia foliascens*	Collection: Red Sea Microbial association: LMA	Symbiont genome: *S. spongiarum* (Gao et al., 2014)
*Cliona varians*	Collection: Atlantic Ocean Microbial association: LMA	Sponge transcriptome (Riesgo et al., 2014b)
*Cymbastela concentrica*	Collection: Australia Microbial association: LMA	Microbial metagenome/metaproteome (Thomas et al., 2010; Fan et al., 2012; Liu et al., 2012) Symbiont genome: δ-proteobacterium (Liu et al., 2011)
*Geodia barretti*	Collection: North Sea Microbial association: HMA	Microbial metatranscriptome (Radax et al., 2012)
*Haliclona cymaeformis*	Collection: Pacific Ocean Microbial association: LMA	Symbiont genomes: sulfur-oxidizing bacterium (Tian et al., 2014)
*Petrosia ficiformis*	Collection: Mediterranean Sea Microbial association: HMA	Sponge transcriptome (Riesgo et al., 2012, 2014a)
*Rhopaloides odorabile*	Collection: Australia Microbial association: HMA	Microbial metagenome (Fan et al., 2012, 2013)
*Stylissa carteri*	Collection: Red Sea Microbial association: LMA	Sponge genome and transcriptome (Ryu et al., 2016) Microbial metatranscriptome (Moitinho-Silva et al., 2014; Ryu et al., 2016)
*Xestospongia* spp.	Collection: Red Sea, Caribbean Sea Microbial association: HMA	Sponge genome and transcriptome (Fiore et al., 2015; Ryu et al., 2016) Microbial metatranscriptome (Fiore et al., 2015; Ryu et al., 2016)

*HMA, high-microbial-abundance sponges; LMA, low-microbial-abundance sponges.*

Despite the increase of genetic data on sponge holobionts, functional studies are missing. Research on gene expression (e.g., transcriptomics) is helpful to fill this gap, especially if applied from an experimental approach (e.g., Riesgo et al., 2014b). Also, RNA interference (RNAi) provides a valuable tool to temporary silence specific genes (e.g., Timmons et al., 2001; Franzenburg et al., 2012). In sponges, promising results from RNAi have been obtained in the freshwater sponge *Ephydatia muelleri* and the marine sponge *Tethya wilhelma*, for genes involved in animal development (Rivera et al., 2011). The authors performed RNAi in marine sponges by feeding them with bacteria expressing the double-stranded RNA of the target gene. Emerging methods to generate targeted genome editing -e.g., clustered regularly interspaced short palindromic repeat associated proteins (CRISPR)/Cas9 mediated genome editing (Gaj et al., 2013)- could allow sponge genome modification. CRISPR, together with Cas proteins, is a nucleic-acid based adaptive immunity found in prokaryotes (Wiedenheft et al., 2012) leading to sequence-specific cleavage of viral invading nucleic acids (Barrangou, 2015) by Cas endonucleases (Jinek et al., 2012). The sequence-specific cleavage of invading DNA by CRISPR-associated endonuclease Cas9 has been adopted to edit genomes in bilaterian model systems (Friedland et al., 2013; Gratz et al., 2013; Hwang et al., 2013; Wang et al., 2013). Since then, this method has revolutionized the possibilities to alter the genome of non-model organisms with unprecedented ease and specificity. Recently, CRISPR/Cas9 genome editing was established in two marine organisms: the cnidaria *Nematostella vectensis* (Ikmi et al., 2014) and the echinoderm *Strongylocentrotus purpuratus* (Lin and Su, 2016). Thus, CRISPR/Cas9 mediated genome-editing presents a promising tool to study mechanisms of symbiosis in sponges, but further effort and investment are required.

The availability of genetically tractable sponges would further contribute to revealing host mechanisms of symbiosis. In other models, host immunity seems to mediate microbial interactions. In particular, pattern-recognition receptors (PRRs) sense microbes (both pathogens and symbionts) and either initiate the defense against pathogenic infection or promote microbiota homeostasis (reviewed in Chu and Mazmanian, 2013). Studies in sponge genomic repertoires have identified a collection of extracellular (i.e., scavenger receptor cysteine-rich, SRCR, domain), membrane-bounded (immunoglobulin-like domains), and intracellular (NOD-like receptor, NLR, domains) PRRs (Hentschel et al., 2012). Some results suggest that immunity may mediate microbial recognition in sponges: (i) high expansion of NLR (in *A. queenslandica*, Degnan, 2015), and SRCR (in *Stylissa carteri*, Ryu et al., 2016) in sponge genomes may correspond to the need for a diverse array of PRRs for effective discrimination upon microbial encounter; (ii) a gene codifying for a SRCR-containing protein was overexpressed in individuals of *Petrosia ficiformis* in symbiosis with cyanobacteria vs. aposymbiotic individuals (Steindler et al., 2007); (iii) Ryu et al. (2016) detected different enrichment in immune domains depending on symbiont densities within the mesohyl (i.e., the genomes of LMA sponges *A. queenslandica* and *S. carteri* vs. the HMA sponge, *X. testudinaria*). The picture of sponge immunity is still incomplete and the development of experimental sponge models would allow validating the function of the predicted genes and the role of immunity in mediating symbiosis.

As for the host, the ability to culture and manipulate symbionts is necessary to further understand the mechanisms of host–microbe interactions. But sponge-associated symbionts, revealed by molecular techniques, remain recalcitrant to culture (Schippers et al., 2012; Hardoim et al., 2014). Alternative cultivation efforts are based on diffusion growth chambers (Steinert et al., 2014), longer incubations, and/or on adjusting culture conditions to match the symbiotic metabolic properties revealed from genomic data (Lavy et al., 2014). In recent years, genomic features of the microbiota from several sponge species have been published (**Table 2**). Also, state-of-the-art sequencing and bioinformatics methods allowed the characterization of the genomes of key sponge-associated symbionts: *Cenarchaeum symbiosum* (Hallam et al., 2006), representatives of the Candidate phylum *Poribacteria* (Siegl et al., 2011), a sponge-associated sulfur-oxidizing bacterium (Tian et al., 2014), and different clades of Ca. *Synechococcus spongiarum* (Gao et al., 2014; Burgsdorf et al., 2015). Sponge symbionts show a wide variety of metabolisms, and some hint at physiological interactions with the sponge host (reviewed in Webster and Thomas, 2016). Metagenomes of sponge-associated microbiota also showed an enrichment on ELPs, suggested to be involved in avoiding host consumption (Thomas et al., 2010). In the absence of tractable models, researchers tested their hypothesis by feeding amoebas with recombinant *E. coli* cells expressing sponge-symbiont-derived ELPs; as a result, the recombinant cells escaped digestion (Nguyen et al., 2014). Further sequencing efforts and cultivation of target symbionts will allow further insight into environment-regulated response, chemical characterization or colonization mechanisms and may become a system amenable to genetic manipulation.

## Summary and Conclusion

Research on sponge-associated microbiota adds valuable insights to our understanding of animal-microbiota symbiosis, mainly because of the natural range of symbiosis within this early-divergent phylum. However, the field needs to move from exploratory to mechanistic projects, where state-of-the-art techniques are applied to meaningful experimental design and the symbiosis is manipulated. Although certain infrastructure is required, aquaculture conditions are described for several species and the regeneration capacity of sponges could serve for keeping clone lines in laboratory. Further research and resources should focus on the physiology and microbiology of cultured sponges over the long term. The new techniques for targeted genome editing appear to be the most promising method for investigating the sponge-microbiota symbiosis through host manipulation.

Finally, the most suitable sponge species for experimental models will depend on the specific focus of the study. In terms of host manipulation, *Tethya wilhelma* and *Ephydatia muelleri* seem the most advanced model species, together with *Amphimedon queenslandica*, with a well annotated genome and possibility of aquaria maintenance. Based on adequate performance in aquaculture, *Clathria prolifera, Dysidea avara, Halichondria panicea, Ianthella basta, Ircinia* spp., or *Mycale laxissima* are valuable candidates but they still require comprehensive genomic data on the symbiosis. The cumulative genomic information on their symbiotic communities and possibilities for *in situ* manipulation or aquaculture of other species such as *Aplysina aerophoba, R. odorabile*, or *Xestospongia* sp., (**Table 2**), necessitate further studies to enhance their tractability. Developing more than one model species will produce a more comprehensive view of the mechanisms of symbiosis. The amenability of laboratory sponge models to manipulation would certainly help to identify key players and key functions of the interaction, and their relevance can be further validate by *in situ* studies in different species and environmental conditions. Thus, researchers take advantage of the insights from an experimentally tractable model in combination with the holistic view of the sponge symbiosis in its natural ecological context.

## Author Contributions

All authors listed, have made substantial, direct and intellectual contribution to the work, and approved it for publication.

## Conflict of Interest Statement

The authors declare that the research was conducted in the absence of any commercial or financial relationships that could be construed as a potential conflict of interest.
